# Evaluating the impacts of digital ECG denoising on the interpretive capabilities of healthcare professionals

**DOI:** 10.1093/ehjdh/ztae063

**Published:** 2024-08-12

**Authors:** Stacey McKenna, Naomi McCord, Jordan Diven, Matthew Fitzpatrick, Holly Easlea, Austin Gibbs, Andrew R J Mitchell

**Affiliations:** B-Secur Ltd, City Quays 3, 92 Donegall Quay, BT1 3FE Belfast, N. Ireland; B-Secur Ltd, City Quays 3, 92 Donegall Quay, BT1 3FE Belfast, N. Ireland; B-Secur Ltd, City Quays 3, 92 Donegall Quay, BT1 3FE Belfast, N. Ireland; B-Secur Ltd, City Quays 3, 92 Donegall Quay, BT1 3FE Belfast, N. Ireland; B-Secur Ltd, City Quays 3, 92 Donegall Quay, BT1 3FE Belfast, N. Ireland; The Allan Lab, Jersey General Hospital, St Helier, Jersey; The Allan Lab, Jersey General Hospital, St Helier, Jersey

**Keywords:** Digital ECG denoising, ECG noise interference, ECG interpretation

## Abstract

**Aims:**

Electrocardiogram (ECG) interpretation is an essential skill across multiple medical disciplines; yet, studies have consistently identified deficiencies in the interpretive performance of healthcare professionals linked to a variety of educational and technological factors. Despite the established correlation between noise interference and erroneous diagnoses, research evaluating the impacts of digital denoising software on clinical ECG interpretation proficiency is lacking.

**Methods and results:**

Forty-eight participants from a variety of medical professions and experience levels were prospectively recruited for this study. Participants’ capabilities in classifying common cardiac rhythms were evaluated using a sequential blinded and semi-blinded interpretation protocol on a challenging set of single-lead ECG signals (42 × 10 s) pre- and post-denoising with robust, cloud-based ECG processing software. Participants’ ECG rhythm interpretation performance was greatest when raw and denoised signals were viewed in a combined format that enabled comparative evaluation. The combined view resulted in a 4.9% increase in mean rhythm classification accuracy (raw: 75.7% ± 14.5% vs. combined: 80.6% ± 12.5%, *P* = 0.0087), a 6.2% improvement in mean five-point graded confidence score (raw: 4.05 ± 0.58 vs. combined: 4.30 ± 0.48, *P* < 0.001), and 9.7% reduction in the mean proportion of undiagnosable data (raw: 14.2% ± 8.2% vs. combined: 4.5% ± 2.4%, *P* < 0.001), relative to raw signals alone. Participants also had a predominantly positive perception of denoising as it related to revealing previously unseen pathologies, improving ECG readability, and reducing time to diagnosis.

**Conclusion:**

Our findings have demonstrated that digital denoising software improves the efficacy of rhythm interpretation on single-lead ECGs, particularly when raw and denoised signals are provided in a combined viewing format, warranting further investigation into the impact of such technology on clinical decision-making and patient outcomes.

## Graphical Abstract

**Graphical Abstract:**
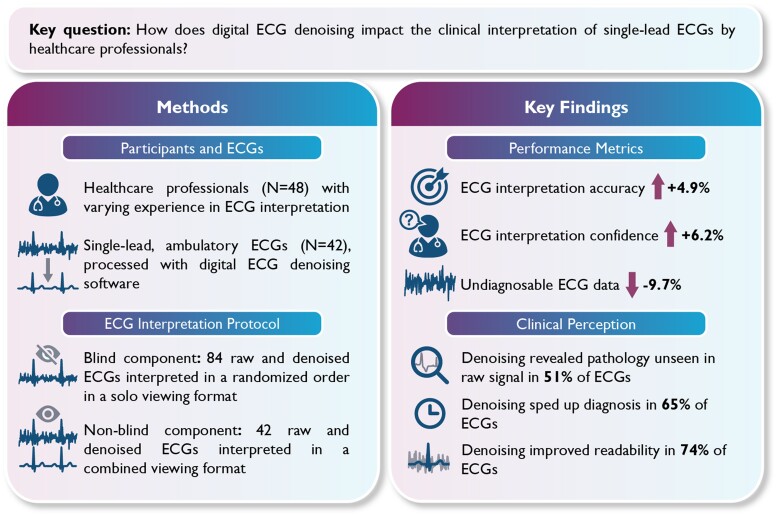

## Introduction

Electrocardiogram (ECG) interpretation is a vital investigative skill that enables the effective triage, diagnosis, and management of numerous medical conditions. Adept interpreters rely on a combination of pattern recognition capabilities and advanced cognitive functions to discern pathological deviations in the ECG, contingent upon the clear identification of diagnostic features.^1,2^ However, this intricate process is challenged by the presence of noise and artefacts originating from non-cardiac sources during signal acquisition. Artefacts that mimic the morphology of pathological features can simulate non-existent arrhythmias and cardiac abnormalities, including atrial fibrillation, ventricular tachycardia, and myocardial ischemia, resulting in erroneous diagnoses and potentially inappropriate interventions.^3–6^ Furthermore, excessive noise interference can distort or obscure the underlying signal, rendering significant portions of recordings clinically unactionable. This can potentially result in missed or delayed diagnoses, a problem that is exacerbated in the ambulatory setting.^7,8^ Implementing effective ECG denoising strategies within clinical workflow is crucial to reduce diagnostic error and mitigate the need for repeat investigation.

In accordance with established consensus standards, medical ECG devices utilise bandpass filters with adjustable frequency cutoffs in conjunction with pre-configured notch filters implemented through analogue components or integrated digital algorithms.^9–12^ Although effective against persistent contaminants of predictable frequency, conventional noise-reduction approaches have shown limited efficacy against dynamic interference sources, such as electromyographic noise and motion-derived artefacts, which vary considerably in frequency and morphology.^13^ Additionally, standard filtering methods have themselves been identified as significant sources of error during ECG interpretation, particularly when improper bandwidth settings are used.^14,15^ Notably, low-pass filtering can reduce the amplitude of Q, R, and S waves, which are essential for diagnosing left ventricular hypertrophy, while high-pass filters can induce shifts in the ST segment that simulate or remove evidence of transient ischaemia.^16–19^

Recent advancements in digital ECG filtering techniques hold promise for overcoming the limitations of conventional filtering approaches.^20,21^ However, there is a notable absence of literature evaluating their impact on the interpretive abilities of healthcare professionals, making it difficult to justify their integration within clinical workflow. While many studies have assessed filtering efficacy using mathematical measures of signal distortion on synthetically noisy signals^22,23^ or by assessing the accuracy of automated feature detection algorithms,^24^ these provide limited insight into their real-world clinical implications. Others have employed small groups of expert cardiologists to evaluate signal interpretability before and after denoising.^25,26^ Nevertheless, these findings lack generalizability across the broad range of ECG-related healthcare professions and experience levels encountered in practice. Additionally, these studies overlook the crucial aspect of assessing ECG interpreters’ perceptions towards the technology, which is essential for its adoption in routine clinical practice to be successful.^27^

The primary aim of this study was to assess the impact of digital denoising on the clinical interpretation of single-lead ECGs and, secondly, to gauge healthcare professionals’ perception towards the technology. Single-lead ECGs prevalent in portable and consumer ECG devices are used in tracking dynamic track heart rate metrics and diagnosing rhythm disturbances, such as atrial fibrillation, particularly in ambulatory settings. However, their clinical utility is often compromised by noise-related issues that hinder interpretation, making effective denoising crucial to maximize the value of single-lead ECG data. By systematically evaluating the impacts of denoising on clinical interpretation, we aim to highlight the critical role it plays in improving the reliability and diagnostic value of single-lead ECGs, ultimately benefitting the healthcare experts who rely on these devices for cardiac assessment.

## Methods

### ECG signals and processing software

**Figure 1:**
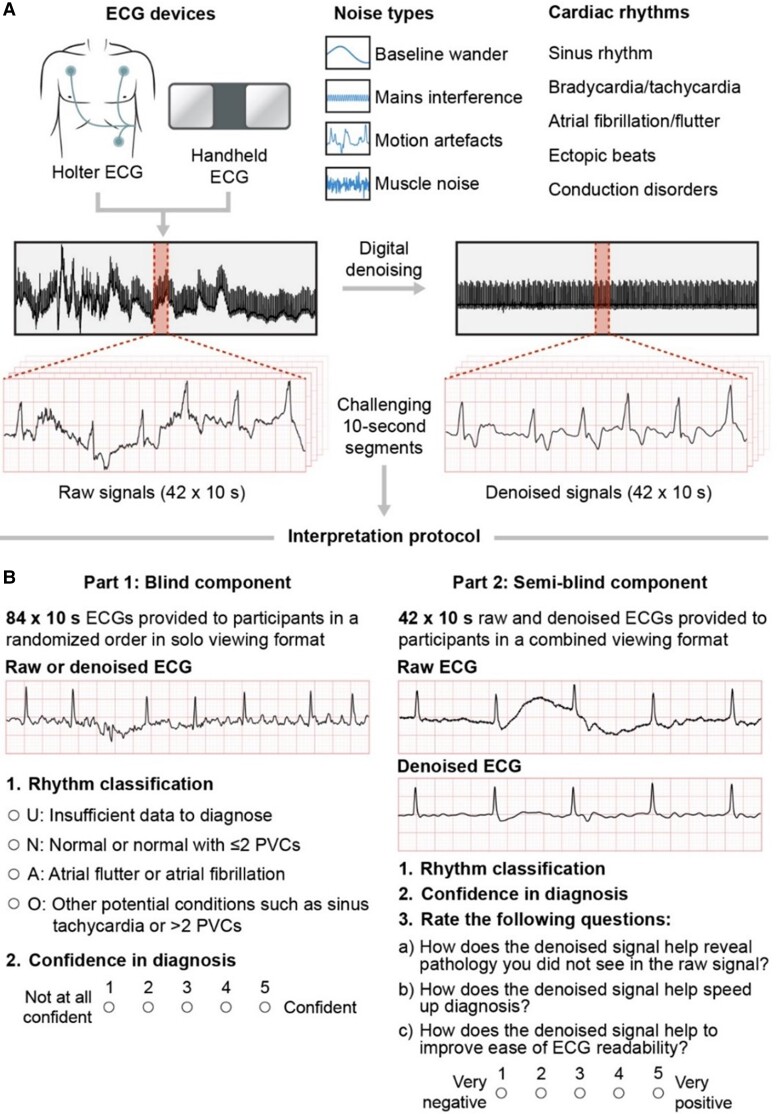
Overview of (*A*) the ECG devices, noise types, and cardiac rhythms included within the single-lead ECG interpretation test set and (*B*) the two-part ECG interpretation protocol. PVC, premature ventricular contraction.

A set of 42 ECG signals was retrospectively selected from proprietary ECG databases recorded by Jersey General Hospital and B-Secur Ltd on two different devices: (i) Bittium® Faros (Oulu, Finland) ambulatory ECG monitors in lead I/II configuration; and (ii) a proprietary single-lead handheld ECG device (*Figure 1A*). To ensure adequate representation of clinical scenarios relevant to single-lead ECG device application, signals were chosen to encompass a range of ECG waveform characteristics, variable sources and intensities of real noise, and a variety of rhythm abnormalities, including atrial fibrillation, atrial flutter, ectopic beats and rhythms, and conduction disorders (*Table 1*).

**Table 1:** Sources and characteristics of the ECG test set signals (*n* = 42)

**ECG device**	
Holter monitor (lead I/II)	36 (86%)
Handheld device	6 (14%)
**Cardiac rhythm**	
Normal sinus rhythm	9 (21%)
Atrial fibrillation or atrial flutter	12 (29%)
Ectopic rhythms	18 (43%)
Heart block (1st, 2nd, or 3rd degree)	3 (7%)
**Noise type**	
Baseline wander	12 (29%)
Powerline interference	9 (21%)
Motion artefacts	8 (19%)
Muscle noise	34 (81%)

The 42 raw signals were extracted and processed using HeartKey® (Belfast, UK) software,^28,29^ generating 42 corresponding denoised ECG signals (84 signals in total). HeartKey is a cloud-based ECG processing platform that employs a series of iterative, logic-based digital filters for denoising, including a mains subtraction filter with adaptive harmonic estimation to cancel interference at 50/60 Hz, a low-pass filter to remove noise above the standard 40 Hz ambulatory cut-off, and a baseline and smoothing filter featuring dynamic components to address non-stationary noise interference. Challenging 10-s segments from each of the corresponding signals were manually selected for the ECG interpretation protocol.

### Baseline survey

A clinical work assessment survey was conducted to investigate the characteristics of recruited participants encompassing factors, such as gender, medical profession, experience and training in ECG interpretation, frequency of ECG interpretation, roles and responsibilities within the ECG workflow, and perceived challenges related to noise in clinical settings.

### ECG interpretation protocol

A two-part interpretation protocol consisting of sequential blinded and semi-blinded components was devised to assess the impact of signal denoising on clinical ECG rhythm interpretation capability (*Figure 1B*). In part one, each of the 84 signals (42 raw, 42 denoised) were independently assessed in a blinded and randomised order, ensuring participants were unaware of whether the signal being assessed was raw or denoised. The rhythm classification categories reflect the primary classes of arrhythmia that can be diagnosed using single-lead ECG and are similar to those used in the PhysioNet Computing in Cardiology 2017 Challenge.^30^ Participants were asked to classify each 10-s ECG strip as one of the following: (i) N: normal or normal with ≤2 PVCs; (ii) A: atrial fibrillation or atrial flutter; (iii) O: other potential cardiac conditions, such as sinus tachycardia or >2 PVCs; or (iv) U: undiagnosable or insufficient data to be confident of the diagnosis. Following this diagnosis, participants assigned confidence scores for the rhythm diagnosis on a five-point graded scale, ranging from 1 (‘not at all confident’) to 5 (‘confident’).

In the second part, the 48 corresponding raw and denoised ECGs were presented in a combined viewing format to enable comparative signal assessment. Participants were instructed to provide rhythm classifications and confidence scores, as described previously. Additionally, participants were asked to provide a subjective rating on a scale of 1 (‘very negative impact’) to 5 (‘very positive impact’) for three statements assessing the perceived impact of signal denoising on clinical ECG workflow for each of the 42 combined ECG strips. Participants completed the assessment using an online Google Form, requiring an estimated 3 h to finish. An example of the interpretation tasks can be found in the supplementary document (see Supplementary material online, *Figures S1* and *S2*).

### Participant recruitment

The study included healthcare professionals aged over 18 who were either undergoing or had completed their medical training. Only those who routinely interpret ECGs as part of their clinical duties were considered eligible for participation. Details of the study were advertised on social media between August and November 2022. Interested healthcare professionals were instructed to submit their CVs for initial eligibility screening. In some instances, cover letters were also requested to provide additional context on the applicants’ experience with ECG. Recruited participants provided informed consent and received financial remuneration upon completion of the interpretation tasks. The remuneration amount for each participant was determined on a sliding scale considering their expertise and experience levels.

### Reference ECG interpretations

The reference diagnosis for each of the 48 signals was determined through consensus agreement using the test annotations provided by the three most experienced consultant cardiologists. In cases where discrepancies arose, a fourth independent cardiologist possessing over 30 years of expertise in ECG interpretation intervened to provide the definitive diagnosis. The fourth cardiologist had access to both the raw and denoised ECG signals, as well as the annotations provided by the other cardiologists.

### Statistical analyses

Data analysis and visualisation were performed using the Scipy, Numpy, and Pandas packages in Python version 3.11.^31–33^ Participant characteristics were summarized using descriptive statistics, with nominal variables presented as counts and means with standard deviations. Accuracy was calculated as the percentage of test annotations that matched the rhythm classification of the reference ECG. Mean values for rhythm classification accuracy, interpretation confidence, and proportion of undiagnosable (‘U’) annotations were calculated for each participant, stratified by categorical variables (interpretation format, experience cohort), and presented alongside the corresponding confidence interval (CI). Shapiro–Wilks tests were performed to examine the normality of the data distribution, followed by a paired sample Wilcoxon signed rank test for group comparisons. The Spearman correlation coefficient was used to examine the correlation between rhythm classification accuracy and confidence scores. All *P*-values were adjusted for multiple comparisons using a post-hoc Bonferroni correction and were considered statistically significant at *P* < 0.05. Descriptive statistics and testing results are provided in the supplementary document (see Supplementary material online, *Tables S1–S6*).

## Results

### Participant characteristics

We recruited 48 healthcare professionals equally distributed across three experience cohorts based on the total number of years spent in an ECG-related role: junior (<5 years), experienced (5–10 years), and senior (>10 years). *Table 2* summarises the characteristics of the participants and their responses to the clinical work assessment survey. The mean number of years spent in an ECG-related role for those in the junior, experienced, and senior experience cohorts was 1.6 (±1.2) years, 6.8 (±2.0) years, and 19.1 (±8.3) years, respectively. Of the total cohort, 19 (40%) were male, and most were located in the UK (65%) and Jersey (27%). Cardiac physiologists (56%) constituted the largest professional group, alongside various other professional cohorts, ranging from junior doctors (17%) to consultant cardiologists (8%).

**Table 2:** Characteristics of study participants and responses to the baseline clinical work assessment survey

	Total (*n* = 48)	Junior cohort (*n* = 16)	Experienced cohort (*n* = 16)	Senior cohort (*n* = 16)
**Years of ECG experience**				
Mean (SD)	9.2 (±8.9)	1.6 (±1.2)	8.1 (±5.5)	17.8 (±8.6)
**Gender**				
Male	19	5	7	7
Female	29	11	9	9
**Location**				
UK	31	8	13	10
Jersey	13	8	1	4
Ireland	2	—	1	1
Other	2	—	1	1
**Medical profession**				
Consultant cardiologist	4	—	1	3
Trainee cardiologist	3	—	2	1
Cardiac physiologist	27	7	10	10
Cardiac nurse	3	1	—	2
GP	3	—	3	—
Junior doctor	8	8	—	—
**ECG device experience**				
12-lead ECG	29	10	9	10
Ambulatory ECG	9	3	3	3
Both	10	3	4	3
**Frequency of ECG interpretation**				
Daily	39	12	12	15
Weekly	8	4	3	1
Monthly	1	—	1	—
**ECG interpretation responsibility**				
Filtering/prioritizing	3	—	1	2
Initial diagnosis	23	11	7	5
Confirming diagnosis	16	4	5	7
All	6	1	3	2
**Estimated % of ECGs in medical practice that contain substantial noise and must be repeated**				
<10%	4	—	2	2
10%	28	8	7	13
25%	13	6	6	1
50%	3	2	1	—
**Typical noise observed during ECG recording**				
Motion artefacts	20	5	9	6
Muscle artefacts	18	7	4	7
Mains interference	5	3	1	1
All	5	1	2	2

The majority of participants (81%) routinely interpreted ECGs on a daily basis as part of their clinical responsibilities, with the primary tasks being to provide an initial diagnosis (60%) or confirm an existing one (46%). Familiarity with different ECG device types varied across the cohort: 81% had experience with 12-lead ECGs, 40% with ambulatory ECG devices, and 21% with both. The majority of participants (92%) reported encountering substantial noise contamination requiring a repeat investigation in ≥10% of ECGs acquired during clinical practice, with muscle noise and motion-induced artefacts being the most frequently observed sources of interference.

### ECG rhythm classification accuracy

**Figure 2:**
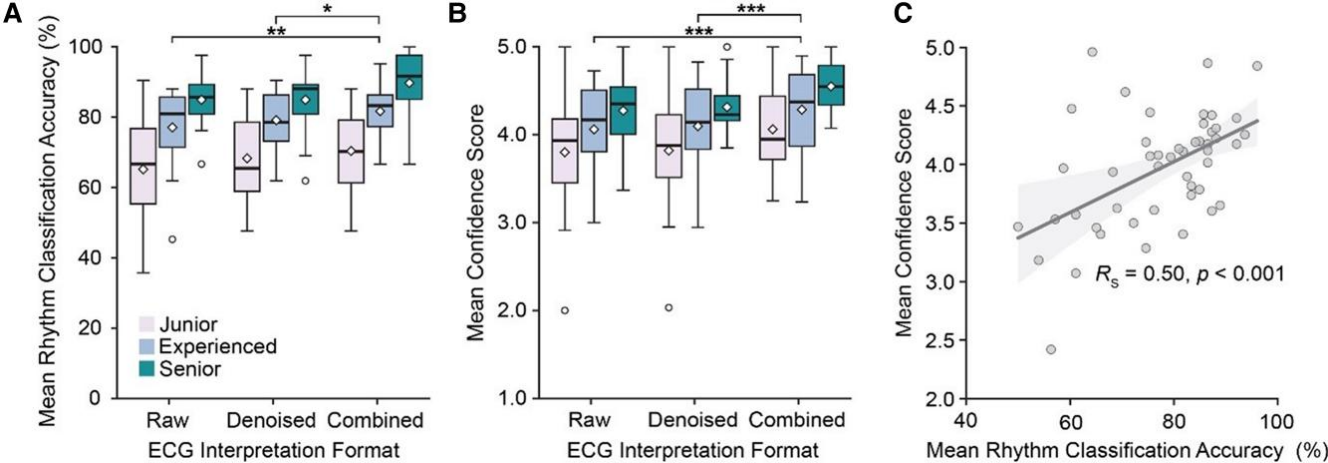
Box-plot showing the distribution of mean rhythm classification accuracies (*A*) and mean confidence scores (*B*) for participants across experience cohorts and ECG interpretation formats. Paired sample Wilcoxon signed-rank test with Bonferroni correction used for multiple comparisons: *P* < 0.05 (*); *P* < 0.005 (**); *P* < 0.0005 (***). Box edges denote the upper and lower quartile boundaries, with whiskers extending to 1.5 times the interquartile range. The median is displayed as a horizontal bar, the mean as a diamond, and outlier values as individual points. Linear regression analysis with 95% confidence interval bands (*C*) shows the relationship between mean confidence score and mean rhythm classification accuracy for each participant across all signals (*R*s = Spearman correlation coefficient).

The combined viewing format yielded the highest mean rhythm classification accuracy of 80.6% (95% CI 77.0–84.2) across all experience cohorts, marking a modest, but statistically significant, mean improvement of 4.9% and 3.1% over the individual raw (75.7% (95% CI 71.5–79.9), *P* = 0.0087) and denoised (77.5% (95% CI 73.9–81.0), *P* = 0.047) signal interpretation formats (*Figure 2A*). These improvements were relatively consistent across experience cohorts, with junior, experienced, and senior groups demonstrating mean interpretation accuracy increases of 5.2%, 4.6%, and 4.8%, respectively, compared to the raw interpretation format. Although the denoised format exhibited a slight mean accuracy increase of 1.8% over the raw format, this was not statistically significant (*P* = 0.574). Additionally, rhythm classification accuracy was positively correlated with ECG experience level, with the mean participant accuracy for the senior cohort [86.6% (95% CI 84.0–89.1)] being significantly greater than that of the experienced [79.3% (95% CI 76.6–82.0), *P* < 0.001], and junior [68.0% (95% CI 64.0–71.9), *P* < 0.001] cohorts.

### ECG interpretation confidence

Participants were generally confident in their interpretations, with the majority of scores falling within the higher confidence categories: 2477 (44.5%) were rated as 5, 1805 (32.8%) as 4, 975 (17.7%) as 3, 244 (4.4%) as 2, and 28 (0.51%) as 1. Lower confidence scores (1 or 2) were infrequent, potentially indicating that interpretations of low diagnostic confidence were assigned as ‘U’ (549, 9.1% of all annotations).

Inter-group confidence trends were comparable to those observed for rhythm classification accuracy. When considering all experience cohorts, the combined signal interpretation format produced a mean confidence score of 4.30 (95% CI 4.16–4.43), representing a percentage increase of 6.2% and 5.4% over the individual raw (4.05 (95% CI 3.88–4.22), *P* < 0.001) and denoised (4.08 (95% CI 3.91–4.24), *P* < 0.001) formats, respectively (*Figure 2B*). Across all signals, the senior cohort demonstrated the highest confidence in their diagnoses, with a mean participant confidence score of 4.38 (95% CI 4.27–4.49), while the junior cohort exhibited the least [3.89 (95% CI 3.71–4.08), *P* < 0.001]. Mean confidence score improvements were similar across cohorts, with mean increases of +6.8% for juniors, +5.6% for experienced participants, and +6.3% for seniors in the combined viewing format compared to raw. The Spearman correlation coefficient of 0.50 (*P* < 0.001) indicated a moderate and statistically significant positive correlation between mean rhythm classification accuracy and mean confidence score (*Figure 2C*).

### Proportion of undiagnosable ECG data

**Figure 3:**
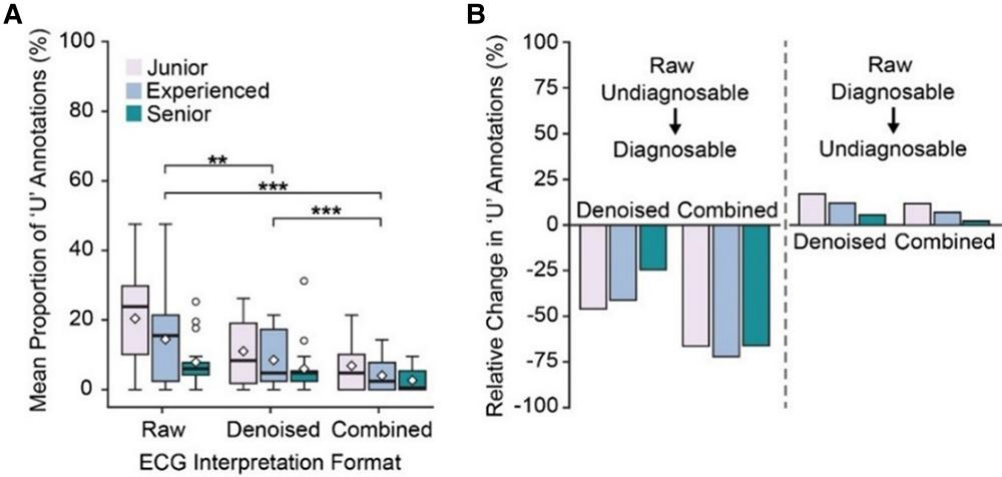
Box-plot showing the mean proportion of ‘U’ annotations for participants across ECG interpretation formats (*A*). Paired sample Wilcoxon signed-rank test with Bonferroni correction used for multiple comparisons: *P* < 0.05 (*); *P* < 0.005 (**); *P* < 0.0005 (***). Box edges denote the upper and lower quartile boundaries, with whiskers extending to 1.5 times the interquartile range. The median is displayed as a horizontal bar, the mean as a diamond, and outlier values as individual points. Histogram showing the relative change in ‘U’ annotations in denoised and combined viewing formats (*D*).

Signal denoising had a profound impact on reducing the number of undiagnosable (‘U’) annotations (*Figure 3A* and *B*). Over the three experience cohorts, the mean proportion of ‘U’ annotations per participant for signals in the raw format [14.2% (95% CI 10.5–17.9)] was significantly higher compared to the denoised [8.5% (95% CI 6.0–11.0), *P* < 0.001] and combined [4.5% (95% CI 2.8–6.2), *P* < 0.001] interpretation formats, corresponding to a relative ‘U’ annotation proportion decrease of 47.1% and 68.3%, respectively. The mean proportion of undiagnosable ECG data decreased with interpreter experience, with the junior cohort [12.7% (95% CI 9.27–16.2)] exhibiting a higher mean proportion of ‘U’ annotations compared to the experienced [9.0% (95% CI 6.0–12.0), *P* = 0.29) and senior cohorts (5.5% (95% CI 3.6–7.5), *P* = 0.002). Reductions in the mean proportion of undiagnosable data were also comparable across cohorts, with mean percentage decreases of 66.7% for juniors, 72.2% for experienced participants, and 65.8% for seniors in the combined viewing format compared to raw.

**Figure 4:**
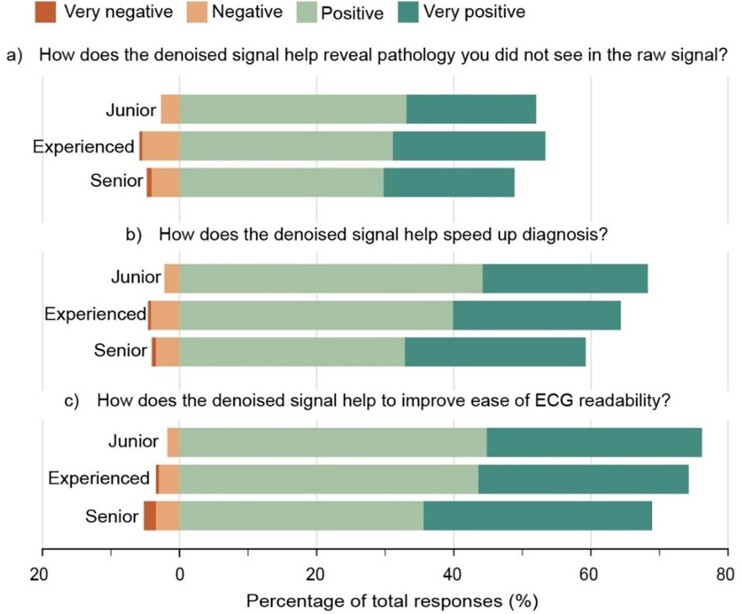
Responses to subjective questions assessing perceived clinical impact of digital ECG denoising. Percentage of responses to each of the three questions in part two of the interpretation protocol. Responses of ‘no impact’ have been omitted for clarity.

Out of the 287 signals annotated as ‘U’ in the raw interpretation format, 203 (36.3%) received diagnoses of ‘N’, ‘A’, or ‘O’ in the denoised format and 231 (41.0%) in the combined format (*Table 3*). The rhythm classification accuracy of annotations changing from ‘U’ when viewed raw to a diagnosis of ‘N’, ‘A’, or ‘O’ once viewed in the denoised (77.0% (changed) vs. 77.5% (total)) or combined (72.0% (changed) vs. 80.6% (total)) viewing formats was lower, but comparable to that of the entire annotation test set. Conversely, of the 5499 annotations of ‘N’, ‘A’, and ‘O’ when viewed in the raw format, 87 (1.6%) of the corresponding signals were annotated as ‘U’ when viewed in the denoised format and 35 (0.6%) when in the combined format.

**Table 3:** Comparison of the changes in the proportion and accuracy of diagnosable annotations (‘N’, ‘A’, or ‘O’) between interpretation formats over the entire cohort

Raw diagnosis (*n*)	Denoised or combined diagnosis	Change in ‘U’ annotations (*n*)	Relative change in diagnosable annotations (%)	Accuracy of changed annotations (%)
Undiagnosable: ‘U’ (287)	Denoised diagnosable: ‘N’, ‘A’ or ‘O’	−203	37.3	77.0
	Combined diagnosable: ‘N’, ‘A’ or ‘O’	−231	68.2	72.0
Diagnosable: ‘N’, ‘A’, or ‘O’ (1729)	Denoised undiagnosable: ‘U’	87	−6.9	—
	Combined undiagnosable: ‘U’	35	−11.6	—

### Clinical perception of digital ECG denoising

Participants had a predominantly positive perception of digital denoising as it relates to the three aspects of clinical ECG workflow that were assessed (*Figure 4*). When comparatively evaluating the raw and denoised signals together in the combined interpretation format, participants stated that the denoised signal had a ‘positive (4)’ or ‘very positive (5)’ impact in 51% of responses to statement 1 (revealing unseen pathology), 65% of responses to statement 2 (speeding up ECG diagnosis), and 74% of responses to statement 3 (improving ease of ECG readability). Importantly, signal denoising was perceived as ‘negative (2)’ in less than 3.7% of responses to each statement, with instances of a ‘very negative (1)’ occurring in less than 0.5% of responses.

## Discussion

Deficiencies in the ECG interpretation abilities of healthcare professionals are a longstanding concern within the medical community. Major errors have been reported in up to 33% of ECG interpretations, and as many as 11% of these errors lead to inappropriate patient management.^34,35^ Even among cardiologists, who are widely regarded as the reference standard for definitive ECG diagnoses, interpretation accuracy can vary considerably, ranging from 49% to 92%.^36^ A recent survey has also highlighted low levels of diagnostic confidence in a diverse cohort of medical professionals, with only 12% feeling comfortable when performing independent ECG interpretation.^37^ Consequently, numerous studies have been conducted to investigate the sources of interpretive error across different medical professions and clinical settings^38,39^ in addition to assessing the efficacy of interventive measures, including educational initiatives,^40^ training tools,^41^ and the use of automated interpretation software,^42^ aimed at addressing these issues. However, despite the established association between noise and diagnostic errors in the ECG, our study is the first to directly evaluate the impact of digital denoising software on the interpretive capability of a diverse cohort of healthcare professionals.

Our results show that the denoising of single-lead ECG signals with an advanced, cloud-based platform afforded participants modest improvements in rhythm classification accuracy and diagnostic confidence while significantly reducing the proportion of undiagnosable data. Consistent with previous research, the participants with greater experience demonstrated higher accuracy and confidence in ECG interpretation.^43^ The positive correlation observed between interpretation accuracy and diagnostic confidence also aligns with prior research, suggesting that healthcare professionals who are more confident in their interpretations tend to achieve higher accuracy.^39,44^ These findings support the view that confidence reflects interpretive proficiency and suggest that denoising may have a synergistic effect, where enhanced signal clarity contributes to both improved interpretive accuracy and increased confidence.

Importantly, as the scale of improvements in rhythm classification accuracy, confidence score, and proportion of undiagnosable annotations was largely consistent across the different experience cohorts, this would suggest that healthcare professionals of all training levels could potentially benefit from using digital denoising platforms to aid the interpretive process. In practical terms, our findings indicate that the implementation of such technology within clinical workflow could potentially reduce the need for repeat investigations by maximising the proportion of actionable ECG data while simultaneously reducing the likelihood of patient mismanagement through erroneous diagnoses, ultimately leading to improved patient outcomes.

A key finding of this study is the importance of presenting raw and denoised ECGs together in a combined viewing format to maximise the benefits of signal denoising. Existing practice guidelines emphasize the need for transparent disclosure of filtering parameters and caution against inappropriate filter use to preserve waveform fidelity.^10,45–48^ However, they do not address the potential impacts of comparative signal evaluation pre- and post-denoising, suggesting that its significance has been overlooked. We postulate that the provision of the raw, unfiltered waveforms alongside denoised signals provides interpreters with additional contextual information to aid in the identification of noise and artefacts that may not be readily discernible in isolation. Furthermore, the comparative approach enables interpreters to effectively cross-validate ECG features, thereby identifying any potential loss or distortion of diagnostic information during denoising, ultimately contributing to improved accuracy and confidence in diagnosis.

For digital health technologies to be successfully integrated within clinical practice, it is essential to address the perceptions and concerns of the healthcare professionals that will utilize them.^27^ If a technology is seen to improve the efficiency of clinical workflows by streamlining diagnostic processes or reducing workload, it is more likely to be accepted.^49^ Conversely, if the potential benefits are deemed insubstantial or significant barriers to use exist, such as extensive training requirements or poor compatibility with existing systems, healthcare professionals may be reluctant to change their practices. Based on the overwhelmingly positive perception of signal denoising observed in this study, coupled with the growing ease of software integration within digital health infrastructures via cloud-based approaches,^50^ we anticipate that such advanced signal denoising platforms could be easily integrated within current clinical workflows and accepted by healthcare professionals with minimal resistance.

This study possesses several limitations. Firstly, despite efforts to ensure representation of a variety of signals with diverse rhythms and noise burdens, logistical constraints restricted the inclusion to a small number of ECG signals in the test set, which is unlikely to reflect the diversity encountered in practice. Secondly, we must also acknowledge a selection bias towards signals with significant noise contamination to reflect the challenging ambulatory use conditions of single-lead ECG devices, resulting in poorer quality recordings compared to those seen in other clinical scenarios. Thirdly, the proprietary denoising platform was not compared with other established techniques for medical ECG filtering. As filter type and parameters impact the fidelity of the denoised signal, this may limit the generalizability of our findings. The relatively small number of recruited participants and heterogeneous representation across different healthcare professions may also limit generalizability. Lastly, although participants perceived the impacts of signal denoising as positive, the study did not generate quantitative data to substantiate these claims.

## Conclusion

This study has demonstrated the efficacy of advanced signal denoising software in reducing the proportion of single-lead ECG data deemed undiagnosable due to noise interference while simultaneously enhancing the interpretive rhythm classification accuracy and confidence of ECG-based healthcare professionals. Crucially, the benefits were markedly improved when both raw and denoised signals were displayed concurrently in a combined viewing format, thereby enabling a comparative feature evaluation, an observation we feel is not adequately reflected in contemporary literature or medical guidelines. Future studies involving larger professional cohorts and more diverse clinical ECG representation are warranted to further validate these findings and determine direct, measurable impacts on clinical decision-making and patient outcomes.

## Ethics statement

This study was conducted in accordance with the Declaration of Helsinki. Proprietary data were collected at B-Secur Ltd. (Belfast, UK) and the Jersey General Hospital (St Helier, Jersey). Patient data were deidentified and managed in line with the European General Data Protection Regulation. Enrolled participants provided informed consent prior to study participation.

## Supplementary Material

ztae063_Supplementary_Data

## Data Availability

The deidentified datasets generated and analysed during the current study are available from the corresponding author on reasonable request.
