# Supplementary material for: Evaluating the impacts of digital ECG denoising on the interpretive capabilities of healthcare professionals
Source: Eur Heart J Digit Health. 2024 Aug 12;5(5):601–10. doi: 10.1093/ehjdh/ztae063 (PMC11417490; doi:10.1093/ehjdh/ztae063)
Supplement: ztae063_Supplementary_Data [file ztae063_supplementary_data.docx]

Supplementary Information


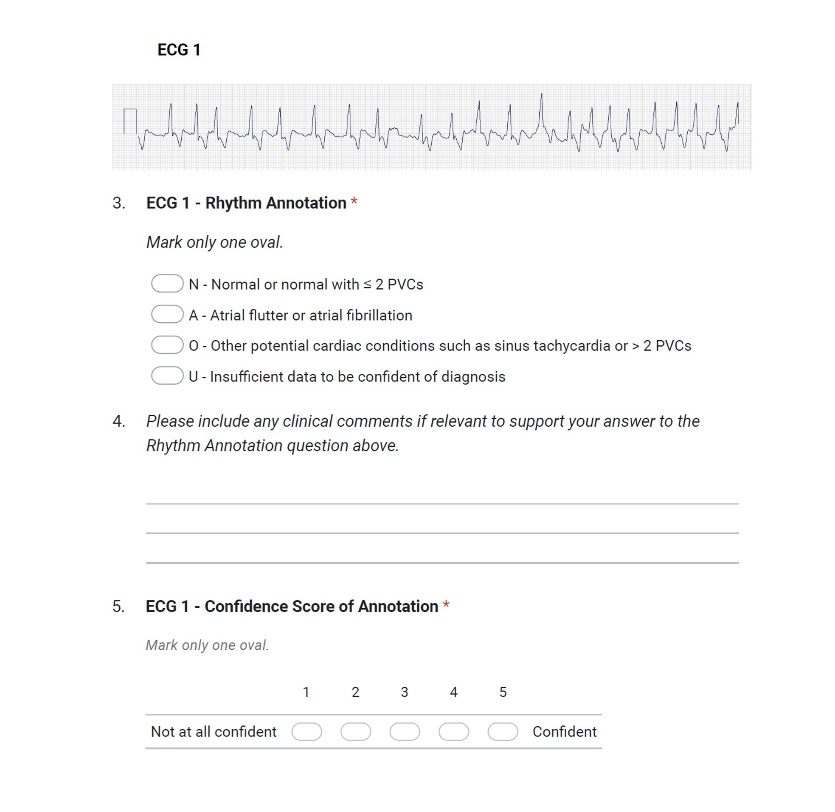


Figure **S1**. An example of the blind component of the interpretation protocol (84 tasks total).


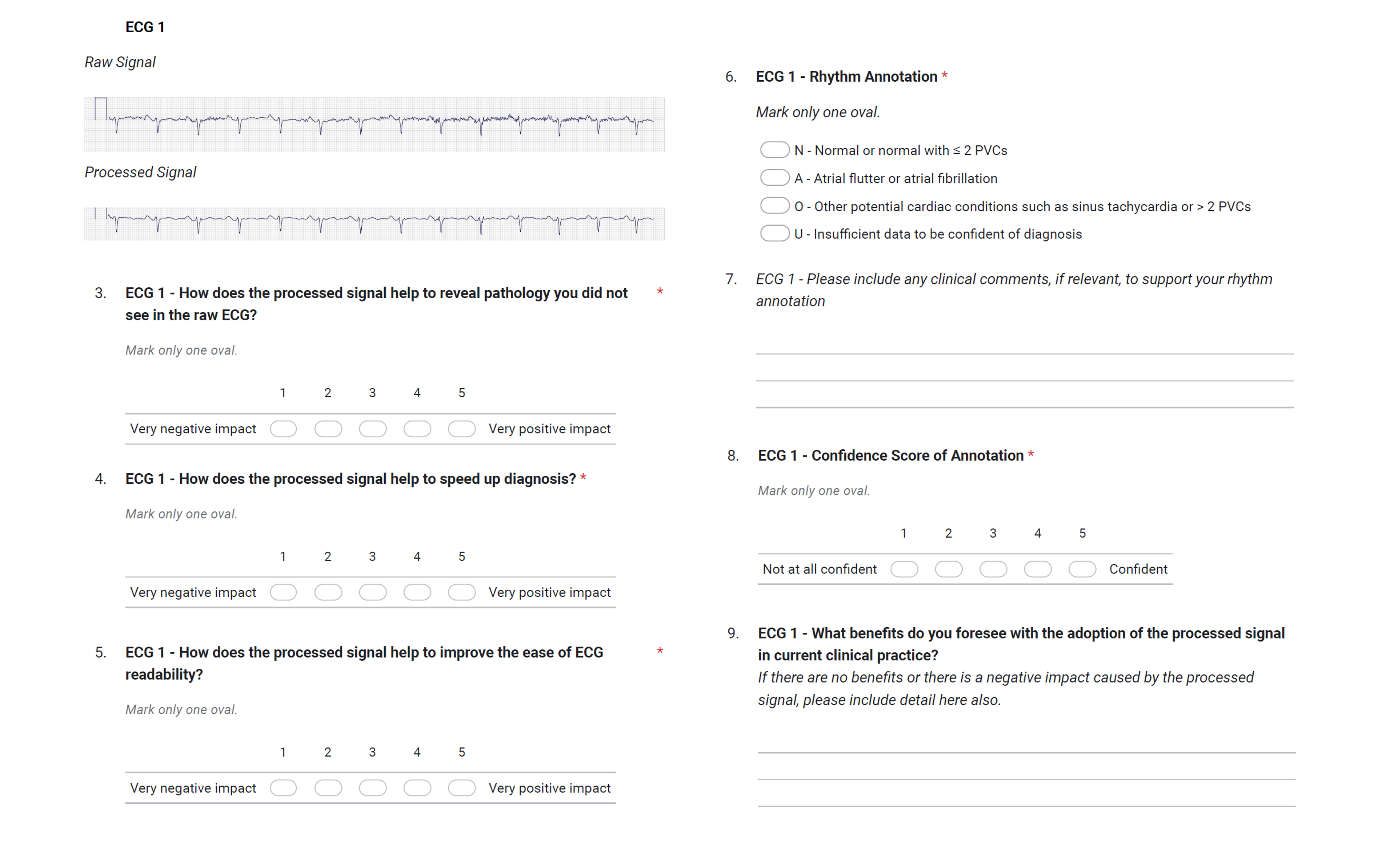


Figure **S2**. An example of the semi-blind component of the ECG interpretation protocol (42 tasks total).

**Mean Participant Rhythm Classification Accuracy**

Table **S1**. Descriptive statistics for participant rhythm classification accuracy analyses.

| **Format** | **Experience** | **Mean (%)** | **SD (%)** | **95% CI (%)** | **Median (%)** | **Range (%)** |
| --- | --- | --- | --- | --- | --- | --- |
| Raw | Junior | 65.2 | 16.1 | 56.6 - 73.7 | 66.7 | 35.7 - 90.5 |
|  | Experienced | 77.1 | 11.3 | 71.1 - 83.1 | 81.0 | 45.2 - 88.1 |
|  | Senior | 85.0 | 7.8 | 80.8 - 89.1 | 85.7 | 66.7 - 97.6 |
|  | **All** | **75.7** | **14.5** | **71.5 – 79.9** | **81.0** | **35.7 – 97.6** |
| Denoised | Junior | 68.3 | 12.4 | 61.7 - 74.9 | 65.5 | 47.6 - 88.1 |
|  | Experienced | 79.2 | 8.6 | 74.6 - 83.8 | 78.6 | 61.9 - 90.5 |
|  | Senior | 85.0 | 9.3 | 80.0 - 89.9 | 88.1 | 61.9 - 97.6 |
|  | **All** | **77.5** | **12.2** | **73.9 – 81.0** | **78.6** | **47.6 – 97.6** |
| Combined | Junior | 70.4 | 12.2 | 63.9 - 70.2 | 70.2 | 47.6 - 88.1 |
|  | Experienced | 81.7 | 7.9 | 77.5 - 83.3 | 83.3 | 66.7 - 95.2 |
|  | Senior | 89.7 | 8.9 | 85.0 - 91.7 | 91.7 | 66.7 – 100 |
|  | **All** | **80.6** | **12.5** | **77.0 – 84.2** | **83.3** | **47.6 - 100** |

Table **S2**. Wilcoxon signed-rank test with Bonferroni correction results for group comparisons on rhythm classification accuracy.

| **Group Comparison** | ***p*-value** | **Corrected *p*-value** | **Significance** |
| --- | --- | --- | --- |
| Raw vs Denoised | 0.19146 | 0.57438 | Not significant |
| Raw vs Combined | 0.00290565 | 0.00871695 | Significant |
| Denoised vs Combined | 0.0157657 | 0.0472971 | Significant |
| Junior vs Experienced | 2.91849e-05 | 8.75547e-05 | Significant |
| Junior vs Senior | 6.37526e-08 | 1.91258e-07 | Significant |
| Experienced vs Senior | 4.33842e-05 | 0.000130153 | Significant |

**Mean Participant Confidence**

Table **S3**. Descriptive statistics for participant confidence score analyses.

| **Format** | **Experience** | **Mean** | **SD** | **95% CI** | **Median** | **Range** |
| --- | --- | --- | --- | --- | --- | --- |
| Raw | Junior | 3.8 | 0.69 | 3.43 - 4.17 | 3.93 | 2.00 - 5.00 |
|  | Experienced | 4.06 | 0.51 | 3.79 - 4.33 | 4.17 | 3.00 - 4.73 |
|  | Senior | 4.28 | 0.45 | 4.04 - 4.51 | 4.35 | 3.37 - 5.00 |
|  | **All** | **4.05** | **0.58** | **3.88** | **4.14** | **2.00 - 5.00** |
| Denoised | Junior | 3.82 | 0.69 | 3.45 - 4.19 | 3.88 | 2.03 - 5.00 |
|  | Experienced | 4.1 | 0.54 | 3.81 - 4.39 | 4.14 | 2.95 - 4.83 |
|  | Senior | 4.32 | 0.32 | 4.15 - 4.48 | 4.23 | 3.85 - 5.00 |
|  | **All** | **4.08** | **0.57** | **3.91** | **4.17** | **2.03 - 5.00** |
| Combined | Junior | 4.06 | 0.5 | 3.79 - 4.33 | 3.95 | 3.25 - 5.00 |
|  | Experienced | 4.29 | 0.49 | 4.02 - 4.55 | 4.38 | 3.24 - 4.90 |
|  | Senior | 4.55 | 0.3 | 4.39 - 4.71 | 4.55 | 4.08 - 5.00 |
|  | **All** | **4.3** | **0.48** | **4.16** | **4.38** | **3.24 - 5.00** |

Table **S4**. Wilcoxon signed-rank test with Bonferroni correction results for group comparisons on participant confidence scores.

| **Group Comparison** | ***p*-value** | **Corrected *p*-value** | **Significance** |
| --- | --- | --- | --- |
| Raw vs Denoised | 0.474231 | 1 | Not significant |
| Raw vs Combined | 9.41287e-06 | 2.82386e-05 | Significant |
| Denoised vs Combined | 5.25036e-06 | 1.57511e-05 | Significant |
| Junior vs Experienced | 0.0551143 | 0.165343 | Not significant |
| Junior vs Senior | 5.26589e-06 | 1.57977e-05 | Significant |
| Experienced vs Senior | 0.0402369 | 0.120711 | Not significant |

**Mean Participant Proportion of Undiagnosable Data**

Table **S5**. Descriptive statistics for proportion of undiagnosable data analyses.

| **Format** | **Experience** | **Mean** | **SD** | **95% CI** | **Median** | **Range** |
| --- | --- | --- | --- | --- | --- | --- |
| Raw | Junior | 20.4 | 14 | 12.9 - 27.9 | 23.8 | 0.0 - 47.6 |
|  | Experienced | 14.4 | 13.4 | 7.3 - 21.6 | 15.5 | 0.0 - 47.6 |
|  | Senior | 7.9 | 7 | 4.2 - 11.6 | 6 | 0.0 - 26.2 |
|  | **All** | **14.2** | **8.3** | **10.5 - 17.9** | **10.7** | **0.0 - 47.6** |
| Denoised | Junior | 11 | 9.5 | 6 - 16.1 | 8.3 | 0.0 - 26.2 |
|  | Experienced | 8.5 | 8.3 | 4.1 - 12.9 | 4.8 | 0.0 - 21.4 |
|  | Senior | 6 | 8 | 1.7 - 10.2 | 4.8 | 0.0 - 33.3 |
|  | **All** | **8.5** | **4.8** | **6.0 - 11.0** | **4.8** | **0.0 - 33.3** |
| Combined | Junior | 6.8 | 7.8 | 2.7 - 11.0 | 4.8 | 0.0 - 21.4 |
|  | Experienced | 4 | 4.7 | 1.5 - 6.5 | 2.4 | 0.0 - 14.3 |
|  | Senior | 2.7 | 3.7 | 0.7 - 4.6 | 0 | 0.0 - 9.5 |
|  | **All** | **4.5** | **2.4** | **2.8 – 6.2** | **2.4** | **0.0 - 21.4** |

Table **S6**. Wilcoxon signed-rank test with Bonferroni correction results for group comparisons on participant proportion of undiagnosable data.

| **Group Comparison** | ***p*-value** | **Corrected *p*-value** | **Significance** |
| --- | --- | --- | --- |
| Raw vs Denoised | 3.36924e-05 | 0.000101077 | Significant |
| Raw vs Combined | 3.72887e-07 | 1.11866e-06 | Significant |
| Denoised vs Combined | 0.000602923 | 0.00180877 | Significant |
| Junior vs Experienced | 0.0970028 | 0.291008 | Not significant |
| Junior vs Senior | 0.000722673 | 0.00216802 | Significant |
| Experienced vs Senior | 0.0207255 | 0.0621765 | Not significant |
